# Cancers of unknown primary origin (CUP) are characterized by chromosomal instability (CIN) compared to metastasis of know origin

**DOI:** 10.1186/s12885-015-1128-x

**Published:** 2015-03-19

**Authors:** Jonas Vikeså, Anne Kirstine H Møller, Bogumil Kaczkowski, Rehannah Borup, Ole Winther, Ricardo Henao, Anders Krogh, Katharina Perell, Flemming Jensen, Gedske Daugaard, Finn C Nielsen

**Affiliations:** 1 Center for Genomic Medicine, Rigshospitalet, University of Copenhagen, Blegdamsvej 9, DK-2100 Copenhagen Ø, Denmark; 2 Department of Oncology, University of Copenhagen, Blegdamsvej 9, DK-2100 Copenhagen Ø, Denmark; 3 Bioinformatics Centre, Department of Biology and Biotech Research and Innovation Centre, University of Copenhagen, DK-2200 Copenhagen, Denmark; 4 Department of Radiology Copenhagen University Hospital, Rigshospitalet, Blegdamsvej 9, DK-2100 Copenhagen Ø, Denmark; 5 Technical University of Denmark (DTU), DK-2800 Lyngby, Denmark

**Keywords:** Carcinoma of unknown primary, Chromosome instability

## Abstract

**Background:**

Cancers of unknown primary (CUPs) constitute ~5% of all cancers. The tumors have an aggressive biological and clinical behavior. The aim of the present study has been to uncover whether CUPs exhibit distinct molecular features compared to metastases of known origin.

**Methods:**

Employing genome wide transcriptome analysis, Linear Discriminant Analysis (LDA) and Quadratic Discriminant Analysis (QDA), we defined the putative origins of a large series of CUP and how closely related a particular CUP was to corresponding metastases of known origin. LDA predictions were subsequently used to define a universal CUP core set of differentially expressed genes, that by means of gene set enrichment analysis was exploited to depict molecular pathways characterizing CUP.

**Results:**

The analyses show that CUPs are distinct from metastases of known origin. CUPs exhibit inconsistent expression of conventional cancer biomarkers and QDA derived outlier scores show that CUPs are more distantly related to their primary tumor class than corresponding metastases of known origin. Gene set enrichment analysis showed that CUPs display increased expression of genes involved in DNA damage repair and mRNA signatures of chromosome instability (CIN), indicating that CUPs are chromosome unstable compared to metastases of known origin.

**Conclusions:**

CIN may account for the uncommon clinical presentation, chemoresistance and poor outcome in patients with CUP and warrant selective diagnostic strategies and treatment.

**Electronic supplementary material:**

The online version of this article (doi:10.1186/s12885-015-1128-x) contains supplementary material, which is available to authorized users.

## Background

Cancers of unknown primary origin (CUPs) are a heterogeneous group of cancers with variable clinical and histological features for which no primary site of the tumor can be identified despite an extensive diagnostic work-up [1,2]. CUPs accounts for 3-5% of all cancer diagnoses and about 85% of the patients have a very poor prognosis [3]. Although a primary tumor cannot be identified in about two-thirds of the cases, CUPs are generally considered to represent metastases. The elusive origin may partly be related to limitations in our diagnostic procedures, but it may also indicate that CUPs exhibit distinct biological features compared to metastasis of known origin [4].

The prevalent model of metastasis is that cells from a primary tumor invade the local environment and spread to distant locations. Metastases may derive from more or less differentiated cancer cells at different stages of tumor growth and this may provide a substantial heterogeneity in the clinical presentation and nature of metastases. Although micrometastases are enriched in cells expressing stem cell markers, macrometastases share many similarities to the primary tumor, so newly settled cancer stem cells not only self-renew, but also foster differentiated colonies of cancer cells [5]. Because metastases retain some of the characteristics of the primary cancer, transcriptome signatures have been employed to depict the origin of CUPs.

It is currently unknown if CUPs exhibit particular genetic and phenotypic characteristics compared to metastases of known origin. The challenge in addressing this problem is obviously that CUPs per definition are of unknown origin. To circumvent this problem, we generated a molecular signature that could classify a wide number of known primary tumor classes and their metastases with high accuracy.

We used the expression signature to classify the CUPs and included a Quadratic Discrimination Analysis (QDA) to generate an outlier score depicting how closely related a particular sample is to the different kinds of primary tumors. Subsequently, we used the LDA predicted classification to make a paired analysis comparing CUPs to their equivalent metastasis of known origin (MOKO), to define a CUP core set of differentially expressed genes that could provide leads to the molecular pathology of CUPs.

We demonstrate that CUPs exhibit a number of distinct molecular features that distinguish them from conventional metastasis. CUP gene expression signatures are more distantly related to their predicted primary tumor classes than signatures of metastases known origin, and they exhibit an inconsistent expression of conventional cancer biomarkers. CUPs are enriched in BRCA1, ATM and CHEK2 DNA damage and homologous recombination repair networks suggesting that CUPs are chromosome unstable and this was corroborated by the demonstration of signatures of chromosome instability (CIN) in CUPs. The results indicate that CUPs may warrant selective diagnostic and therapeutic strategies distinct from the current platinum based and organ specific therapy.

## Methods

### Gene expression profiles for tumor classification

Expression profiles of more than 2400 tumor samples were downloaded from the Gene Expression Omnibus (GEO) (http://www.ncbi.nlm.nih.gov/geo/) (Testset: GSE2109, GSE7307, GSE6004, GSE6764, GSE10135, GSE2328, GSE13471, GSE7392 and GSE12606) (Validationset: GSE2109, GSE3325, GSE5764, GSE5764, GSE5787, GSE7307, GSE7476, GSE7553, GSE10245, GSE11151, GSE14762, GSE15471, GSE17537, GSE19826, GSE19829, GSE20565) or generated from samples collected and processed at our own facility at Rigshospitalet (ArrayExpress, E-MTAB-3222). Finally thyroid samples were retrieved from ArrayExpress data base (accession E-MEXP-2442). The specific identifiers of the samples are depicted in Additional file 1: Table S1. The numbers refer to the GSM number in the GEO profile data base and the name (e.g. breast) refers to the biopsy tissue site. The material comprised 15 classes of carcinomas from thyroid, lung, stomach, colon/rectum, pancreas, bile duct/gallbladder, liver, kidney, urinary tract, prostate, breast, ovary, endometrium, cervix uteri, testis cancer and 1 group of malignant melanomas and finally a group with pooled normal tissue samples from various organs that was included in order to allow detection of samples without sufficient tumor tissue. The 16 tumor classes were selected to represent the most frequently identified primary tumor sites in CUP patients at autopsy, and primary tumors that are difficult to distinguish by IHC tools alone due to the lack of specific IHC markers (e.g. upper GI) and/or tumor dedifferentiation. Each tumor class contained the most common histological subtypes. Sample IDs are indicated in the enclosed Additional file 1: Table S1. The pathology descriptions were reviewed in order to group the samples into tumor classes and this ultimately resulted in a set of 1466 expression profiles from well-defined primary tumors (1299) and normal tissue (167) (Additional file 1: Table S1). The classifier was tested on an independent validation set including 641 tumor samples (391 primary tumors and 250 metastases) from all 16 tumor classes (Additional file 1: Table S1).

### CUP patients and samples

CUP patients were consecutively enrolled between November 2004 and September 2010 for diagnostic work-up and treatment. Newly diagnosed CUP patients were referred to the Department of Oncology (Rigshospitalet) for further diagnostic work-up and treatment. All patients had a biopsy-proven metastatic cancer and had undergone diagnostic work-up at the referral hospitals. At the Department of Oncology at Rigshospitalet further diagnostic work-up was performed including revision of biopsies by an experienced pathologist, new biopsies and further imaging procedures. A schematic representation of the CUP patients and the inclusion of samples are shown in Additional file 2: Figure S1. Patients were included when the diagnostic work-up, as recommended by the European Society of Medical Oncology (ESMO) [6], failed to identify a primary site of origin. At least two ultra-sonography-guided biopsies – one for histopathological work-up and one for gene expression profiling – were obtained from all patients. Patients, in whom a putative a primary tumor site eventually was identified in the diagnostic work-up period, were treated according to national guidelines whereas most CUP patients were offered platin/taxane-based regimens as first-line treatment. The study was approved by the Danish RegionH ethical committee and patients had given their written informed consent and have consented for publication and disclosure of clinical data.

### Microarray analysis and expression values

Total RNA was isolated, labeled and hybridized as described [7]. Cell files were pre-processed using the *Robust multi-chip average* (RMA) method [8] and evaluated for quality parameters with the Simpleaffy functionality of the *R/Bioconductor* packages. The data sets were filtered to exclude probe sets with Interquartile Range (IQR) below 0.8.

### Tumor classification and outlier analysis

Linear discriminant analysis (LDA) was used for classification as implemented in the R language. Briefly, in LDA the predictive probability of class c given input x is computed using Bayes’ theorem p(c|x) = p(x|c) p(c)/p(x), where p(x|c) is a normal density specific for the class, p(c) the a priori probability of class c and p(x) = sum_c p(x|c) p(c) the density of the input according to the model. Maximum likelihood is used to fit p(x|c) and p(c), c = 1,…,17 on the training data. In order to construct a gene signature for our classifier we used leave‐one‐out cross validation (LOOCV), where for each split, feature selection by F-test were applied prior to LDA. A grid search over p-value cut-offs yielded the cut-off with the optimal LOOCV accuracy. The signature was eventually selected by an F-test using the optimal p‐value cut‐off on the full set of 1466 training samples, resulting in 428 probes (311 unique genes). The performance of this first (428 probe) classifier was then assessed using the independent 641 sample validation set. We merged the original training and validation set and used the found p-value cut-off (giving 641 probes) to generate a second classifier optimized for CUP prediction. The performance of this classifier was assessed using LOOCV. Finally, the LDA classifier was made sex-specific by setting the prior probabilities to zero for sex specific cancers (ovary, cervical and prostate) not occurring and in the sex in question renormalizing the remaining prior probabilities accordingly. A low model density p(x) implied that the input x was not similar to those in the training data. We therefore defined an outlier score OS = –log p(x) and calculated the OS for each sample in the LOOCV loop. We used QDA (individual covariance of normals) rather than LDA (shared covariance of normals) in this step.

### Gene set enrichment analysis

A CUP core list of transcripts was defined by a paired analysis between CUP LDA predictions and corresponding metastasis of known origin. The pairing was done by making a linear model of the data by eliminating the difference between the groups as implemented in the Qlucore Omics Explorer™ software. Analysis of the CUP core lists (up and down) was performed using the Broad Institutes MSig “Compute overlaps for selected genes” function available on the homepage http://www.broadinstitute.org/gsea/msigdb). Gene symbols in the CUP core lists were analyzed for enrichments of Gene Onthology (GO) genesets (C5). CUP core lists were also analyzed for enrichments of gene sets in the cu rated gene set database (C2). The C2 gene set collection is gathered from various online pathway databases, publications from PubMed and knowledge of domains experts (see homepage). A filter setting was added to both analyses to show only gene sets with FDR q-value below 0.01. GSEA on predefined gene sets were performed using the Broad Institute GSEA v2 software. The expression data matrix was preprocessed in the Qlucore Omics Explorer™ software and expression values were normalized within LDA predictions. The data set was analyzed employing 1000 permutations with all the default standard settings of the GSEA v2 software. Hierarchical cluster analysis was performed and visualized using the Qlucore Omics Explorer™ software. All hierarchical clusters are build using average linkage and heat map was generated based on mean m = 0, variance 1 normalization.

## Results

### CUP patients and tumor classification

Sixty eight consecutive CUP patients were enrolled in the study, but since eleven samples did not meet the quality criteria the number of CUP samples ended at 57. The histological features of the 57 CUP that underwent expression profiling are summarized in Table 1. During the diagnostic work-up, a possible primary tumor site was eventually identified in 28 of the 57 patients (Additional file 2: Figure S1 and Table 1). Among these 18 samples were in accordance with diagnostic work-up or the *Standard of Reference.*

**Table 1:** **Prediction results in CUP patients**

ID	Sex	Biopsy site	Histology	Path Diag.	Stand of Ref	LDA Pred	Outlier score
14.	F	LN neck	PDC	Lung	Lung (CD)	Lung	975
17.	F	LN neck	Adenoc.	Lower GI	Colon (RD)	Colon	746
22.	M	LN neck	Adenoc.	CUP	Stomach (RD)	Normal	934
23.	M	LN retro	PDC	CUP	Kidney (RD)	Kidney	1085
28.	F	Peritoneum	PDA	Ovary	Ovary (RD)	Ovary	810
31.	F	LN neck	PDA	CUP	Lung (CD)	Stomach	985
34	M	Skin	PDA	Lung	Lung (RD)	Lung	898
39.	M	Liver	Adenoc.	CUP	Pancreas (CD)	Pancreas	1097
40.	F	Liver	Adenoc.	Colon	Colon (RD)	Colon	729
44.	F	Kidney	Carc.	CUP	Bladder (RD)	Bladder	1286
49.	M	LN neck	PDA	Kidney	Kidney (RD)	Kidney	1223
51.	F	LN pelvis	SCC	CUP-SCC	Cervical (RD)	Cervix	828
52.	M	Liver	PDA	CUP	CCC (RD)	CCC	923
53.	M	Liver	Adenoc.	Lung	Lung (RD)	Lung	1047
57.	M	Liver	Adenoc.	CCC	CCC (RD)	HCC	965
66.	F	Liver	PDA	CUP	CCC (RD)	Cervix	1100
70.	M	Peritoneum	Adenoc.	Stomach	Stomach (CD)	Colon	842
74.	M	Leg	Carc.	Adnex tumor	Adnex tumor (RD)	Normal	1010
76.	M	Liver	Adenoc.	Lower GI	Small intestine (RD)	Colon	912
77.	M	LN axilla	PDC	CUP	Lung (CD)	Breast	978
86.	F	LN axilla	Adenoc.	CUP	Lung (RD)	Stomach	1108
88.	F	Peritoneum	Adenoc.	Ovary	Ovary (RD)	Cervix	1033
89.	F	Liver	PDA	CCC	CCC (RD)	CCC	916
90.	F	Peritoneum	Adenoc	Ovary	Ovary (RD)	Ovary	781
92.	M	Liver	Malignant tumor	Angiosarcoma	Angiosarcoma (RD)	Normal	1097
95.	M	Peritoneum	PDC	DSRCT	DSRCT (RD)	Breast	1098
71 + 72	M	Bone + Kidney	PDC	Kidney	Kidney (RD)	Kidney	1096
							1277
75 + 87	F/43	Liver	PDA	CCC	CCC (RD)	CCC	925
							1030
**ID**	**Sex**	**Biopsy site**	**Histology**	**Path Diag.**	**Stand of Ref**	**LDA Pred**	**Outlier score**
11.	F	LN neck	PDA	CUP	CUP (SD)	Ovary	756
13.	F	Peritoneum	PDA	CUP	CUP (NSD)	Pancreas	1193
21.	M	LN neck	PDC	CUP	CUP (NSD)	Breast	1108
26.	F	Skin	PDA	CUP	CUP (NSD)	Breast	971
32.	M	LN neck	PDSCC	CUP-SCC	CUP (NSD)	Normal	926
33.	M	Skin	PDA	CUP	CUP (NSD)	Colon	1098
41.	M	Liver	PDA	Pancreas	CUP (NSD)	Stomach	1040
42.	M	Liver	Adenoc.	CUP	CUP (NSD)	Pancreas	994
43.	F	LN retro	PDA	CUP	CUP (NSD)	Stomach	797
45.	M	Liver	PDC	CUP	CUP (NSD)	Colon	1245
46.	F	Liver	Adenoc.	CUP	CUP (NSD)	Normal	1027
47.	F	Liver	Adenoc.	CUP	CUP (SD)	CCC	932
48.	F	LN neck	PDC	CUP	CUP (NSD)	Ovary	1032
54.	F	Liver	Adenoc.	CUP	CUP (NSD)	Normal	1068
55	F	Liver	Adenoc.	CUP	CUP (NSD)	Normal	962
58.	F	Liver	PDC	CUP	CUP (SD)	CCC	995
61.	M	Liver	Carc.	HCC	CUP (NSD)	CCC	1102
64.	F	LN inguien	PDA	CUP	CUP (SD)	Lung	1168
65.	M	LN neck	PDSCC	CUP-SCC	CUP (NSD)	Breast	929
73.	M	LN retro	PDC	CUP	CUP (NSD)	Normal	1020
78.	F	Lung	Adenoc.	Lower GI	CUP (NSD)	Lung	1062
80.	F	Liver	Adenoc.	CUP	CUP (SD)	CCC	1111
81.	F	Liver	PDA	CUP	CUP (NSD)	Breast	1212
82.	F	Bone	Adenoc.	CUP	CUP (NSD)	CCC	1209
83.	F	Liver	PDA	CUP	CUP (SD)	CCC	1061
91.	F	LN axilla	Adenoc.	CUP	CUP (SD)	Lung	939
93.	M	Bone	PDSCC	CUP-SCC	CUP (NSD)	Breast	940
94.	F	Liver	PDA	CUP	CUP (NSD)	Normal	984
50. + 68	M	Adr gl	PDC	CUP	CUP (NSD)	Stomach	978
						Pancreas	1079

A validation of the LDA predicted diagnoses was performed by comparing with a Standard of Reference (SR). SR was established by an experienced pathologist and two experienced oncologists. In addition to the 23 patients where a primary tumor site was identified (Reference Diagnosis (RD)) within the study period, the Standard of Reference reached a Consensus Diagnosis (CD) in 5 patients based on patient demographics, metastatic pattern, results of clinical and laboratory tests, imaging data and pathologic evaluations (Samples labeled in red). In the 29 remaining CUP labeled in blue, the results from gene expression profiling were compared with clinicopathological features and the predictions were categorized as Supportive (SD) or Non-Supportive (NSD). LN: lymph node; n: neck LN; m: mediastinal LN; a: axilla LN; r: retroperitoneal LN; p: pelvis LN; adr gl: adrenal gland; Adenoc: adenocarcinoma, PDA: poorly differentiated adenocarcinoma; Carc: carcinoma; PDC: poorly differentiated carcinoma; SCC: squamous cell carcinoma; PDSCC: poorly differentiated SCC; CCC: cholangiocarcinoma; HCC: hepatocelluar carcinoma; DSRCT: desmoplastic small round cell tumor.Path Diag: pathological diagnosis; Stand of ref: Standard of reference; LDA pred: Linear discriminant analysis prediction; RD: Reference Diagnosis; CD: Consensus Diagnosis, SD: Supportive Diagnosis; NSD: Non-Supportive Diagnosis.

**Figure 1:**
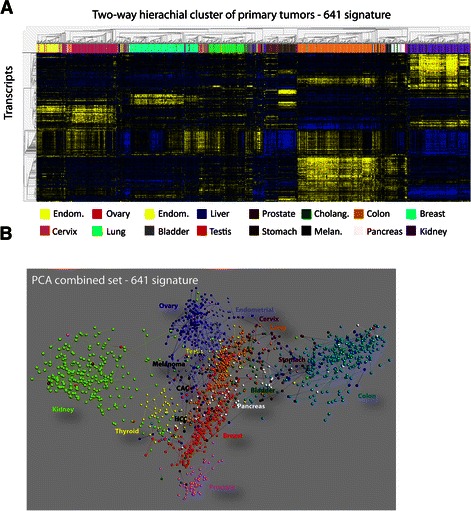
**Hierachial cluster and principal component analysis of tumor classes. A**. Two-way hierachial cluster of 16 tumor classes by the 641 transcript signature. The tumor classes are shown at the top of the cluster and the transcripts are clustered at the left side. **B**. Principal component analysis (PCA) of primary tumors and known metastases based on the signature. The tumor classes are colored and indicated in association with the corresponding tumor samples.

To examine if CUP exhibit particular genetic and phenotypic characteristics compared to metastases of known origin, that could warrant particular diagnostic procedures and treatment, we first generated a transcriptome-based signature that could classify 16 common tumor classes and predict the origins of CUP and metastases of known origin with high accuracy (Detailed in Additional file 1: Table S1). To allow detection of samples without sufficient tumor tissue, a group of normal tissues was also included. Since all CUP data were generated at our facility, we moreover examined a series of primary cancers and metastases from Rigshospitalet to exclude possible site- and batch-specific effects. The cross-validation accuracy during training of a 428 probe sets classifier was 92.2% (Additional file 3: Table S2) and the overall accuracy in the validation set was 90% and 83% for primary tumors and known metastases, respectively (Additional file 3: Table S2). The distribution of variables among the 16 tumor categories is depicted in the heat map (Figure 1). Since we suspected that the low accuracy in some of the classes, e.g. cholangiocarcinoma, was associated with the small number of samples in the training set, and because CUPs were supposed to be compared to metastases of known origin, we subsequently combined the training and validation sets and generated a second classifier, consisting of 641 probe sets (641 classifier). Furthermore a gender correction by renormalizing the prior class probabilities in the test situation was implemented because we noted that tumors from males incorrectly were classified as ovary, cervical and endometrial cancer. The accuracy in primary tumors, known metastases and normal samples of the 641 classifier was 92%, 87% and 89%, respectively (Additional file 3: Table S2) and this classifier was subsequently used for the prediction of CUP. The principal component analysis is shown in Figure 1B and the ten most selective transcripts and their gene ontology for each tumor class are listed in Additional file 4: Figure S2.

**Figure 2:**
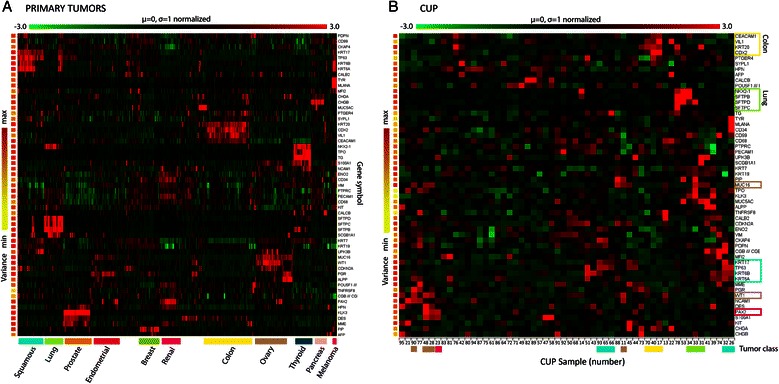
**Patomarkers in primary tumors and CUP.** Probeset Ids for 45 common histopatological markers were collected and used to generate a two-way hierarchal cluster with a selection of primary tumors (Panel **A**) or CUP (Panel **B**). The variance of the individual markers is shown to the left and the scale is indicated at the top of the clusters. Gene symbols are shown to the right and the different tumor classes are shown below ((Panel **A**), primary tumors). For the CUP samples (Panel **B**), groups of markers corresponding to different tumor classes are indicated by the boxes around the gene symbols at the right side of the cluster. The number below the cluster indicated the number of the CUP sample corresponding to the annotation in Table 1.

To provide a systematic overview of the expression of conventional tumor markers in the CUP samples, we also compiled a list of 45 common histopathological biomarkers and depicted their expression in a two-way hierarchal cluster (Figure 2). Whereas, about 85% of the primary cancers exhibited a characteristic expression of their individual histomarkers, only 10 of the 28 (35%) CUP - where a putative primary site was identified and 3 of the 29 (10%) CUP - where the primary site remained unknown - expressed one or more biomarkers at significant levels. The strongest overlap between histopathological markers and the LDA based CUP classifications was observed for CUP predicited as ovary and colorectal cancers, where 4 and 3 samples expressed *WT1* or *CEA/CEACAM5*, respectively. Moreover, 6 samples were positive for *TP63* and 2 samples were positive for surfactant proteins. Finally, one sample was positive for *PAX2* in agreement with the LDA prediction as renal carcinoma. Compared to the primary cancers there was a limited concordance between markers within the same tumor category. Only two of the *WT1* positive cancers were positive for *CA125/MUC16*, and only 3 of the TP63 positive samples expressed *CK17* and *CK5*, characteristic of squamous carcinoma. If the histological markers were combined and used in an LDA based fashion, the concordance with the 641 signature LDA predictions or *Standard of Reference* was about 66% indicating that systematic application of the patomarkers may at least to some extent compensate for the modest predictive power of individual markers.

### QDA based outlier analysis

To determine the similarity between primary cancers, metastases of known origin and CUPs, we employed Quadratic Discriminant Analysis (QDA) to determine the likelihood that a particular sample belonged to one of the predefined tumor classes. Outlier scores were calculated in LOOCV fashion for one sample at a time using all remaining samples i.e. primary tumors and metastases to represent the classes. The outlier scores of the samples from normal tissues are not comparable to the primary tumors and metastases because of the heterogeneity among the many different tissues in the class.

**Figure 3:**
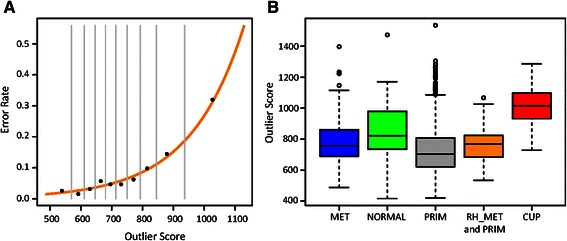
**QDA derived outlier scores in CUP. A)** To determine the relationship between prediction error and outlier scores the primary cancers and metastases were divided into ten bins according to the outlier scores and the error rate was calculated for each bin. Each point represents the error rate plotted versus the median outlier score of the bin. The vertical lines show the span of outlier scores within the bins. The plot shows that higher outlier score translates into higher error rate. We modeled the relationship between outlier scores and prediction error by fitting polynomial function to the data points (the orange line), and the function allows us to estimate the expected error rate for new samples of unknown origin, once their outlier scores have been determined. **B)** Samples from CUP patients tend to have higher outlier scores than other cancer patients. The box plot summarizes the distributions of outlier scores within metastases (MET), primary (PRIM) and CUP tumors. There is a clear tendency for CUP samples to have higher outlier score than metastases and primary cancers. The median outlier score of CUP samples of >1000 suggests the origin prediction error above 30%. On the other hand, most primary cancers and metastases have outlier scores below 800, hence the estimated prediction error from 2-10% (see panel **A**). Since data for CUP and some primary tumors and metastases were generated at Rigshospitalet, the non-CUP samples from Rigshospitalet are presented as separate group (RH_MET and RH-PRIM), this is to show that the shift in outlier scores was not caused by technical bias. Additionally, the normal, non-cancerous tissue group (NORMAL) is included, and shows the whole range of outlier scores.

Based on the results from primary tumors and metastases we plotted the predictive error rates versus the outlier scores and demonstrated a clear relationship between errors and outlier scores (Figure 3). Samples with outlier scores below 800 exhibited less than 10% risk of being erroneous, whereas, outlier scores above 1000 had more than 25% risk of being incorrect. However, even in the high end of outlier scores with only 75% accuracy, prediction is far from random, since we are working with 16 different classes. As shown in the box plot (Figure 3), CUP samples had significantly higher outlier values than primary tumors and metastases. To ensure that the difference was not related to our platform, we compared our own samples of known metastases and primary tumors and observed the same difference. CUPs, moreover, consisted of biopsies that may contain more normal tissue than samples obtained during surgery. We therefore plotted the percentage of normal tissue as estimated from the relative expression of markers of lymphoid, liver, and muscle tissue versus the outlier scores, but observed no correlation between the amount of normal tissue in the biopsies and the outlier scores (Additional file 5: Figure S3). A number of samples that expressed conventional histopathological biomarkers exhibited low scores, but if we compared CUPs where a primary cancer was identified during the clinical processing with CUPs where no primary site could be identified, there was no difference between the outlier scores (mean 991 vs mean 1031, P = 0.24). Taken together, the results demonstrate that CUPs are more distantly related to the predefined tumor classes, than known metastases.

### mRNA Expression and Gene Set Enrichment in CUP

To identify differentially expressed transcripts, we performed a class comparison between CUP and metastases of known origin. The analysis was performed as a paired analysis with respect to the LDA predictions to eliminate differences between tumor classes. Metastases from uterine, testis, prostate, melanoma and thyroid cancers were excluded from the analysis because no CUPs had been allocated to these groups. CUPs predicted as normal tissue were also excluded. Moreover, cholangiocarcinomas were omitted from the calculations because they were not represented in the LDA predicted metastases group. In total 41 CUP and 186 metastases comprising 10 different cancer groups were included in the analysis. To define the most up- and down-regulated CUP transcripts, a cut-off of p < 10^−8^ corresponding to a false discovery rate of q < 1.9*10^−7^ was employed. This resulted in 1550 down- and 1390 up-regulated probe sets corresponding to 1117 and 934 unique annotated genes, respectively. These two lists comprised our CUP core set of differentially expressed transcripts. The 40 most significantly down- or up-regulated mRNAs are shown in Additional file 6: Table S4. The lists of genes was subsequently subjected to a Gene Set Enrichment Analysis (GSEA) using the Broad Institute’s GSEA database (http://www.broadinstitute.org/gsea/msigdb). Initially, we searched for enriched gene ontology terms, and this revealed that up-regulated transcripts were associated with GO-terms (q < 0.01): DNA_INTEGRITY_CHECKPOINT,DNA_DAMAGE_CHECKPOINT,DNA_REPLICATION_INITIATION,DNA_PACKAGING,NEGATIVE_REGULATION_OF_DNA_METABOLIC_PROCESS,CELL_CYCLE_CHECKPOINT;NEGATIVE_REGULATION_OF_DNA_REPLICATION,CHROMATIN_REMODELING,DNA_DAMAGE_RESPONSESIGNAL_TRANSDUCTION. There were no particular enrichments among the down-regulated mRNAs.

To depict CUP enriched molecular pathways, we further examined if the CUP core set exhibited overlaps with the Molecular Signature Database (MSigDB) curated gene sets. Overlaps between the CUP core set (p < 10^−8^) were computed by submission of up- and down-regulated probe sets separately (Table 2). Gene sets consisting of transcripts that were positively correlated to BRCA1, ATM and CHECK2 expression were highly enriched in the up-regulated CUP core set. The down-regulated CUP mRNAs showed fewer significant overlaps but SHEN_SMARCA2_TARGETS_DN gene set, which depict transcripts that are negatively correlated with SMARCA2 expression in prostate cancer was clearly overlapping with the CUP set.

**Table 2:** **Enriched or depleted gene sets in CUPs compared to metastases of known origin**

Up-regulated in CUP				
Gene Set name	Transcripts	Overlap	k/K	p value
**PUJANA_BRCA1_PCC_NERWORK**				
Genes constituting the BRCA1-PCC network of transcripts whose expression positively correlated (Pearson correlation coefficient, PCC > = 0.4) with that of BRCA1	1671	159	0.0952	0.00E + 00
**KINSEY_TARGETS_OF_EWSR1_FLII_UP**				
Genes up-regulated in TC71 and EWS502 cells (Ewing’s sarcoma) upon knockdown of theEWSR1-FLII fusion	1281	133	0.1038	0.00E + 00
**PUJANA_ATM_PCC_NETWORK**				
Genes constituting the ATM-PCC network of transcripts whose expression positively correlated (Pearson correlation coefficient, PCC > = 0.4) with that of ATM	1461	152	0.104	0.00E + 00
**PUJANA_CHEK2_PCC_NETWORK**				
Genes constituting the CHEK2-PCC network of transcripts whose expression positively correlates (Pearson correlation coefficient, PCC > 0.4) with that of CHEK2	782	89	0.1138	0.00E + 00
**DODD_NASOPHARYNGEAL_CARCINOMA_DN**				
Genes down-regulated in nasopharyngeal carcinoma (NPC) compared to the normal tissue.	1375	157	0.1142	0.00E+00
**RODRIGUES_THYROID_CARCINOMA_ANAPLASTIC_UP**				
Genes up-regulated in anaplastic thyroid carcinoma (ATC) compared to normal tissue.	721	93	0.129	0.00+00
**MILI_PSEUDOPODIA_HAPTOTAXIS_UP**				
Transcripts enriched in pseudopodia of NIH/3T3 cells (fibroblast) in response to haptotactic migratory stimulus by fibronectin, FN1	552	74	0.1341	0.00E+00
**RODRIGUES_THYROID_CARCINOMA_POORLY_DIFFERENTIATED_UP**				
Genes up-regulated in poorly diffrentiated thyroid carcinoma (PDTC) compared to normal thyroid tissue.	640	94	0.1469	0.00E+00
**DECOSTA_UV_RESPONSE_VIA_ERCC3_DN**				
Genes down-regulated transcripts in fibrolasts expressing ethier XP/CS or TDD mutant forms of ERCC3 [Gene ID=2071], after UVC irradiation	855	126	0.1474	0.00E+00
**DECOSTA_UV_RESPONSE_VIA_ERCC3_COMMON_DN**				
Common down -regulated transcripts in fibroblasts expressing either XP/CS orTDD mutant forms of ERCC3 [Gene ID=2071], after UVC irradiation	420	64	0.1524	0.00E+00
**OSMAN_BLADDER_CANCER_UP**				
Common down-regulated in blood samples from bladder cancer patients	402	57	0.1418	5.55E-16
**SENUPTA_NASOPHARYNGEAL_CARCINOMA_WITH_LMP1_UP**				
Genes up-regulated in nasopharyngeal carcinoma (NPC) positive for LMP1 [Gene ID=9260], a latent gene of Epstein Barr virus (EBV)	399	56	0.1404	155E-15
**SENUPTA_NASOPHARYNGEAL_CARCINOMA_UP**				
Genes up-regulated in nsopharyngeal carcinoma relative to the normal tissue.	286	46	0.1608	3.33E+15
**PUJANA_XPRSS_INT_NETWORK**				
Genes constituting the XPRSS-Int network: intersection of genes whose expression correlates with BRCA1, BRCA2, ATM, and CHEK2 [Gene ID=672, 675, 472, 11200] in a compendium of normal tissues.	167	34	0.2036	1.21E-14
**Down-regulated in CUP**				
**Gene Set Name**	**Transcripts**	**Overlap**	**k/K**	**p value**
**SHEN_SMARCA2_TARGETS_DN**				
Genes whose expression negatively correlated with that of SMARCA2 [GeneID=6595] in prostate cancer samples	360	73	032028	0.00E+00
**GINESTIER_BREAST_CANCER_ZNF217_AMPLIFIED_DN**				
Genes doen-regulated in non-metastic breast cancer tumors having type 1 amplifications in the 20q13 region; involves ZNF217 [Gene ID=7764] locus only.	336	49	0.1458	7.71E-11

Gene set enrichments among up or down regulated mRNAs in the CUP core set were examined in the molecular signatures database (MSig) among the C2 curated gene sets comprising profiles from chemical and genetic perturbations, canonical pathways, BIOCARTA, KEGG and the reactome collections. The uncorrected p values are indicated. In all cases the false positive discovery rate was set to q < 0.01.

**Figure 4:**
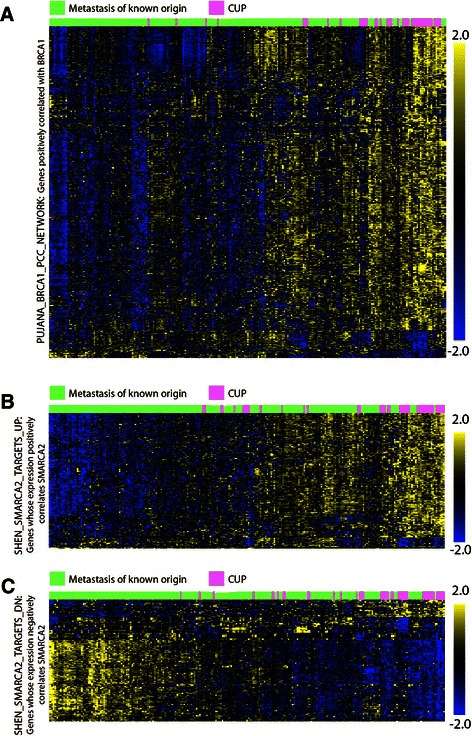
**Two way hierachial clusters of BRCA1 and SMARCA2 networks in metastases and CUP. (A)** The PUJANA_BRCA1_PCC_NETWORK was downloaded from the MSig database (http://www.broadinstitute.org/gsea/msigdb) and used to generate a paired two way hierarchical cluster with known metastases and CUPs. Gene symbols were translated into probe sets and because of the probe set redundancy the data were filtered by a p < 0.001 before clustering. Following filtering 1297 probe sets were included in the clustering. Known metastases are indicated in green and CUP samples are labeled with pink above the cluster. The scale is shown at the right side of the cluster. **(B)** Two-way cluster of the SHEN_SMARCA2_ TARGETS up- and **(C)** downregulated transcripts. The sets consists of 360 down- and 430 up-regulated genes that translated into 772 and 1211 probe sets, respectively. The known metastases are indicated in pink and CUP samples are labeled with green below the cluster. The scale is shown at the right side of the cluster.

To examine the BRCA1 and SMARCA2 pathway networks defined by the SHEN_SMARCA2_TARGET_DN, SHEN_SMARCA2_TARGET_UP and PUJANA_BRCA1_PCC_NETWORK in greater detail, we generated two way clusters using the complete gene sets on our CUP core set (Figure 4). The clusters were based on a paired analysis with respect to their LDA predictions and with the same inclusion criteria, as described above. The SHEN_SMARCA2_TARGET_DN; SHEN_SMARCA2_TARGET_UP and PUJANA_BRCA1_PCC_NETWORK gene symbols were translated into probe sets and to exclude non-functional redundant probe sets, only the probe sets with the 50% highest variance were included. We moreover applied a p-value cut-off of 0.001 to filter probe sets that differed among the two groups (Figure 4). The PUJANA_BRCA1_PCC_NETWORK set of genes consists of 1671 gene symbols that translated into 3897 probe sets. Following filtering 705 probe sets corresponding to 519 up-regulated and 66 down-regulated genes were clustered (Figure 4). From the cluster it is apparent that the BRCA1 profile is strongly enriched in CUP compared to the corresponding metastases. A schematic representation of the BRCA1 and non-homologous repair networks showing the enriched factors is depicted in Additional file 7: Figure S4. Following the same procedure, we subsequently looked at the SMARCA2 networking (Figure 4). The SHEN sets consist of 360 SMARCA2 negatively- and 430 SMARCA2 positively- correlated genes that translated into 772 and 1211 probe sets respectively. In the SMARC2A negatively correlated group, we observed 20 genes that were up-regulated and 95 that were down-regulated in CUP compared to metastases, and amongst the SMARCA2 positive correlated genes we saw 161 up-regulated genes and 19 down regulated after filtering (top 50% variance probes and p < 0.001). Taken together, the GSEA shows that CUPs are characterized by enrichment of the double strand break DNA repair system and the SMARCA2/BRM chromatin dependent remodeling system.

### Chromosome instability in CUP

**Figure 5:**
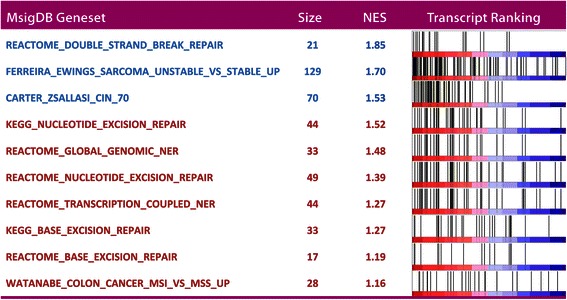
**Signatures of genomic instability in CUPs.** Messenger RNA signatures of chromosomal instability (CIN), DNA double-strand break repair, nucleotide excision repair (NER), base excision repair (BER) and mismatch repair (MMR) in CUPs and MOKO were examined with the Broad Institute GSEA v 2 software. The names of the individual signatures, the number of transcripts and the normalized enrichment scores (NES) are indicated. The right panel depicts the transcript ranking on a colometric scale. With the exception of the CIN signature obtained from [10] all gene lists were retrieved from http://www.broadinstitute.org/gsea/msigdb.

**Figure 6:**
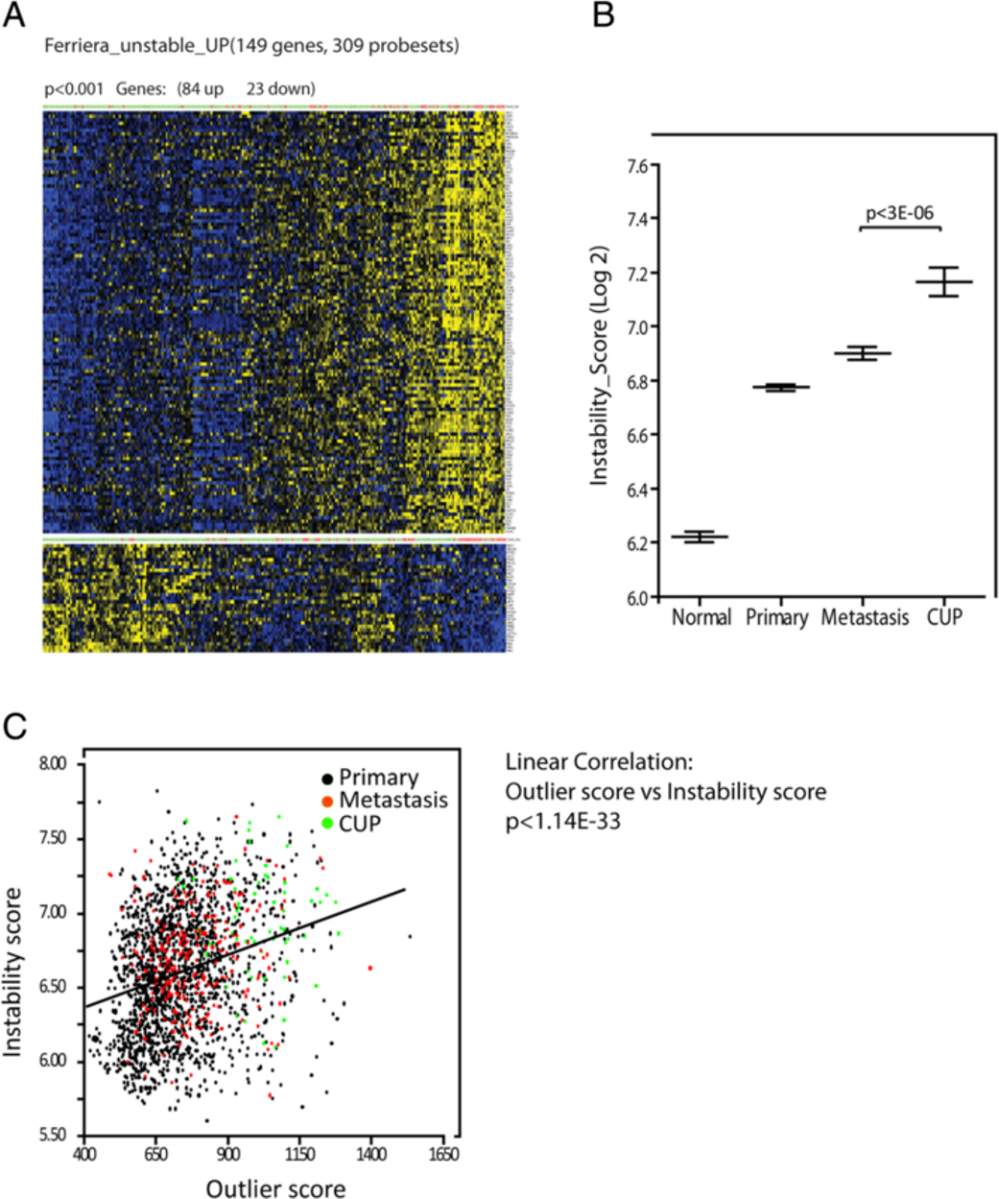
**Chromosomal instability and outlier scores in CUP.** Panel **A**. Two-way hierachial cluster of the Ferreira_Ewing_Sarcoma_Unstable signature in MOKO and CUPs. MOKO and CUPs are indicated by green and pink labels, respectively. Panel **B**. Instability scores in Normal tissues, Primary tumors, MOKO and CUPs. The signature of chromosome unstable sarcoma was employed to generate an instability score calculated as the mean of the expression values from the included probe sets of the signature following variance filtering (206 probe sets). Panel **C**. Linear correlation between outlier scores and instability scores. Primary tumors, metastasis of known origin and CUP are indicated as black, red or green dots, respectively. The lower panel shows the value of the instability scores depicted in a green to red color scale. The p-value of the linear correlation between outlier and instability scores is indicated.

Since the observed enrichment of genes involved in DNA double-strand break repair (Additional file 7: Figure S4) indicated that CUPs were more chromosome unstable than known metastasis and primary cancers, we examined the status of signatures involved in DNA repair and genome instability. Signatures of chromosomal instability (CIN), DNA double-strand break repair, nucleotide excision repair (NER), base excision repair (BER) and mismatch repair (MMR) were included to obtain a complete overview of DNA- repair and stability in CUP (Figures 5 and 6). The predefined gene sets were examined with the Broad Institute GSEA v2 software. The expression data matrix was preprocessed in Qlucore Omics Explorer™ and expression values were normalized within LDA predictions - so the expression values became expressed as a relative value compared to the mean expression of a gene within its group. The data set was analyzed against the 10 selected gene sets (Figure 5) employing 1000 permutations with standard GSEA settings. The most significant scores were observed for the signature of double strand break repair and for signatures of unstable sarcoma [9] and CIN [10]. Moreover, the KEGG signature of NER was enriched but not to a significant level (p = 0.123). The remaining nucleotide excision and mismatch repair signatures were not enriched in CUP and we infer that CUPs primarily distinguishes themselves from metastasis of known origin by signatures of chromosome instability. The signature of chromosome unstable sarcoma was finally employed to generate an instability score providing an index of the chromosomal instability for comparison of normal tissue, primary cancers and metastasis and CUP (Figure 6). The instability score was calculated as the mean of the expression values from the included probe sets of the signature following variance filtering (206 probe sets). As shown in Figure 6 panel B CUP exhibited a significantly higher score than paired metastasis of known origin. Metastases were significantly more chromosomal unstable than primary cancers and as expected normal tissues exhibited very low instability scores. We moreover examined the correlation between outlier scores and instability scores, because chromosome instability is envisioned to promote evolution and phenotypic variations. In a linear correlation analysis including primary cancers, metastasis and CUPs the outlier scores were positively correlated with the chromosomal instability score (p < 7.24E-33; q < 2.16E-30), so we infer that chromosomal instability is likely to be implicated in the phenotypic traits of CUPs.

## Discussion

To examine the biological differences between metastasis of known origin and CUPs, we first developed a robust LDA classifier that could define the most likely origin of a particular CUP on a molecular basis. In line with a number of previous molecular prediction studies [11-25] CUPs were predicted to mainly emanate from bile duct/cholangiocarcinoma, breast, lung and colorectal cancers (Additional file 8: Table S3). Quadratic discrimant analysis (QDA) was subsequently used to calculate the distance of primary tumors, metastases and our CUP samples to the nearest tumor class. In agreement with the acquisition or loss of phenotypic traits compared to their origin, CUPs were more distantly related to the predefined tumor classes than known metastases.

The explanation for the disparity between CUPs and known metastases could obviously be that CUPs were derived from types or subclasses of cancers not represented among our 16 classes, but a number of arguments speak against this. Firstly, autopsy and previous molecular classification studies support that the vast majority of CUPs are likely to originate from the included tumor classes [26]. Secondly, the genetic signature was selected by means of an F-test considering the entire class, so class specific transcripts are supposed to be present even in putative subclasses. Thirdly, high outlier scores were also observed among classes such as colorectal cancers that are not known to contain subclasses. Finally, if a number of CUPs represented rare cancers, the majority of the CUP scores should have overlapped with metastases of known origin. So, taken together, we infer that the observed difference in outlier scores is likely to reflect that CUPs exhibit distinct molecular features.

Attempts to elucidate the molecular biology of CUPs have been hampered by the heterogeneity of the cancers and their elusive origins. Previous studies have indicated that activation of MAPK, cMET and pAKT axes were associated with progression and outcome in CUPs, whereas p21 expression conferred a favorable prognosis [27-29]. Based on the LDA predictions it became, however, possible to define CUP enriched transcripts and molecular pathways in a paired analysis with metastasis of known origin. At the single transcript level one of the most consistently down-regulated factors was early growth response 1 (*EGR1*), which is involved in cell growth and differentiation. Suppressed *EGR1* levels have previously been reported in breast carcinoma [30], glioblastoma [31] and lung [32] cancer, where it was predictive of poor outcome.

The search for gene set enrichments moreover showed that CUPs were enriched in transcripts encoding BRCA1, ATM and CHEK2 DNA damage and homologous recombination repair networks suggesting that CUP are chromosome unstable. This was corroborated by the direct demonstration of mRNA signatures of chromosome instability (CIN) in CUPs. CIN is strongly associated with poor outcome and drug resistance [10], and CIN positive tumors reconcile many of the characteristics of CUP (for review [33]). Previous analyses of the cytogenetic profile of CUPs have also shown that chromosomal changes are frequent and widespread in CUP and that CUPs exhibiting large numbers of chromosomal aberrations have a poor outcome [34]. CUPs are moreover frequently resistant to platinum salts (for review [35] in agreement with the presence of CIN and concurrent up-regulation of the homologous recombination repair [36].

The molecular basis of CIN in sporadic cancers is incompletely understood but oncogene induced collapse of DNA replication forks, leading to DNA double strand breaks and genomic instability is considered an appealing model [37]. CIN is a major driving force for tumorigenesis because it promotes accumulation of transforming genotypes and increases the acquisition of independent phenotypic traits, that may translate into atypical presentations [38]. In agreement with this we observed a clear correlation between the CIN score and the outlier scores that portrays the similarity of the cancers to known groups.

Systemic cancer progression has been proposed to occur via two models. The prevailing model states that cancer progression occurs within the primary tumor before metastatic dissemination of fully malignant cells, whereas the second brings forward that cells disseminate from the primary tumor at an early stage and pursues a parallel and independent progression of metastases (reviewed in [39]). The two models provide a rationale for the observed difference between metastases of known origin and CUPs, because the parallel progression predicts greater disparity between metastatic founders and primary tumor cells than does linear progression. By inference, CIN is likely to facilitate parallel progression by the early accumulation of distinct genetic and epigenetic alterations in the primary tumor and metastases. Moreover, tumor cells are predicted to settle at unconventional sites due to their independent selection and spread before the primary cancer causes clinical symptoms.

## Conclusion

We conclude that CUPs are characterized by chromosome instability, which distinguishes them from metastases of known origin. We propose that CIN and parallel metastatic behavior may be implicated in early dissemination and poor outcome of CUPs.

### Availability of supporting data

Gene expression CEL files are available at Gene Expression Omnibus (GEO) (http://www.ncbi.nlm.nih.gov/geo/) (Testset: GSE2109, GSE7307, GSE6004, GSE6764, GSE10135, GSE2328, GSE13471, GSE7392 and GSE12606) (Validationset: GSE2109, GSE3325, GSE5764, GSE5764, GSE5787, GSE7307, GSE7476, GSE7553, GSE10245, GSE11151, GSE14762, GSE15471, GSE17537, GSE19826, GSE19829, GSE20565) or at ArrayExpress https://www.ebi.ac.uk/arrayexpress/ accession E-MTAB-3222 (samples collected and processed at our own facility). Thyroid samples can be retrieved from accession E-MEXP-2442. The specific identifiers are depicted in Additional file 1: Table S1. Supplemental scripts for the outlier analysis and classifier are available at: http://www.genomic-medicine.dk/recent-papers-and-additional-f/supplemental-files-for-recent-publications/.

## Additional files

Additional file 1:
**Classification and outlier scores of primary tumors, metastasis and CUP.**

Additional file 2: Figure S1. CUP patients and the processing of tumor samples. Sixty-eight CUP patients were consecutively enrolled in the study. In 60 patients the biopsy was obtained during the diagnostic work-up, whereas eight patients received first-line CUP therapy prior to the biopsy. Eleven tumor samples were excluded because the RNA integrity or yield did not meet the required quality criteria. In this way the number of CUP patients ended up at 57. During the diagnostic work a primary tumor site was identified in 23 patients (Table 1) and a consensus diagnosis based on patient demographics, metastatic pattern, results of clinical and laboratory tests, imaging data and pathological evaluations was obtained in five patients as described. In 29 patients the primary tumor site remained unknown.
Additional file 3: Table S2. Prediction of tumor classes with the 428 and 641 classifiers.
Additional file 4: Figure S2. Gene families and tumormarkers A. The 10 most selective transcripts in the 641 classifier for each tumor class. Transcripts were selected by comparison of the indicated tumor class with all other primary cancers and normal tissues and the ten transcripts exhibiting the lowest p value are shown. Due to the small sample number in cholangiocarcinoma and hepatocellular carcinoma, transcripts exhibited p < 0.01 and 10^−4^, respectively. For all other groups p < 10^−10^. Genes marked in red were considered to be almost exclusive to the class. B. Comparison of gene families in the patomarker and 641 classifier gene sets. Gene symbols of the two gene sets were submitted to the Brookhaven gene set enrichment analysis molecular signatures database (http://www.broadinstitute.org/gsea/msigdb) and examined with the Gene Families feature. The diagram shows the percentage of transcripts in each functional category. The 641 classifier transcripts are shown in red bars and the patomarkers are shown in blue bars.
Additional file 5: Figure S3. Percentage of normal cells in CUP biopsies and QDA derived outlier scores. The percentage of normal cells in the CUP biopsies was determined by comparing the levels of liver (*APOA2, ALB*), muscle (*ACTA1*), lymph node (*IGJ, IGHA1, IGKV3-20*) and skin (*KRT2, TYRP1*) specific transcripts in the samples with the expression of the transcripts in corresponding normal tissues where the biopsies were obtained. The number indicates the CUP sample code and samples labeled in yellow were classified as normal tissue. The blue graph shows the corresponding outlier score generated by the QDA analysis.
Additional file 6: Table S4. Forty most up---(red) or down---regulated (blue) mRNAs in CUP compared to metastasis of known origin. Differentially expressed transcripts, were retrieved by a class comparison between CUPs and metastasis The analysis was performed as a paired analysis with respect to the LDA predictions to eliminate differences related to the individual tumor classes. Metastases from endometrial, testis, prostate, melanoma and thyroid cancers were excluded from the analysis because no CUP had been allocated to these groups by the LDA. CUPs predicted as normal tissue were also excluded.
Additional file 7: Figure S4. Schematic representation of CUP enriched BRCA1 DNA damage response and non-homologous end joining repair networks. Diagrams were generated by the Ingenuity software (Ingenuity systems, USA). Up-regulated factors are indicated in red.
Additional file 8: Table S3. LDA predictions in CUP.
