# Supplementary figures and images for: Cancers of unknown primary origin (CUP) are characterized by chromosomal instability (CIN) compared to metastasis of know origin
Source: BMC Cancer. 2015 Mar 19;15:151. doi: 10.1186/s12885-015-1128-x (PMC4404593; doi:10.1186/s12885-015-1128-x)

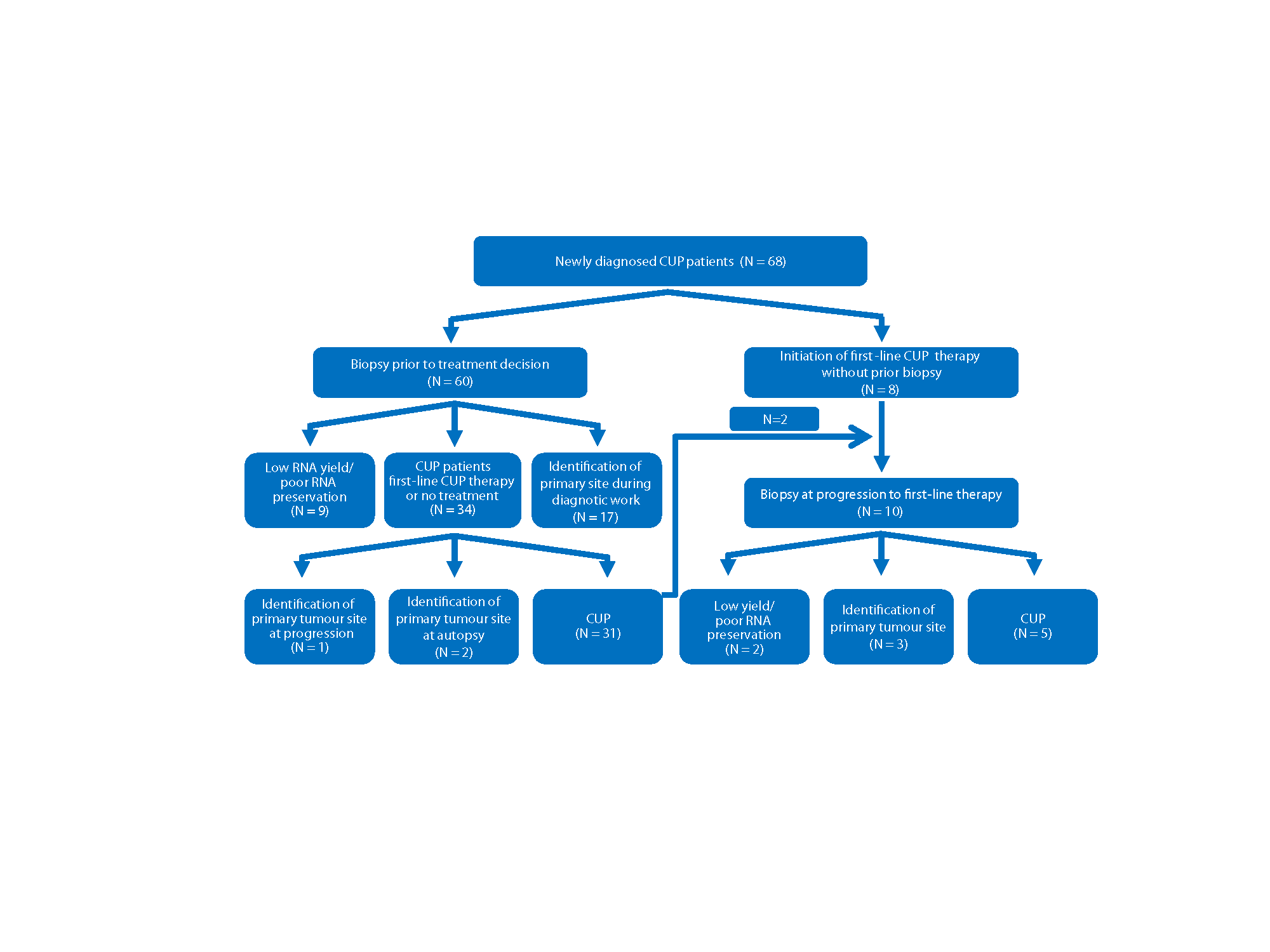

Supplement: Additional file 2: Figure S1. — CUP patients and the processing of tumor samples. Sixty-eight CUP patients were consecutively enrolled in the study. In 60 patients the biopsy was obtained during the diagnostic work-up, whereas eight patients received first-line CUP therapy prior to the biopsy. Eleven tumor samples were excluded because the RNA integrity or yield did not meet the required quality criteria. In this way the number of CUP patients ended up at 57. During the diagnostic work a primary tumor site was identified in 23 patients (Table 1) and a consensus diagnosis based on patient demographics, metastatic pattern, results of clinical and laboratory tests, imaging data and pathological evaluations was obtained in five patients as described. In 29 patients the primary tumor site remained unknown. [file 12885_2015_1128_MOESM2_ESM.tiff]

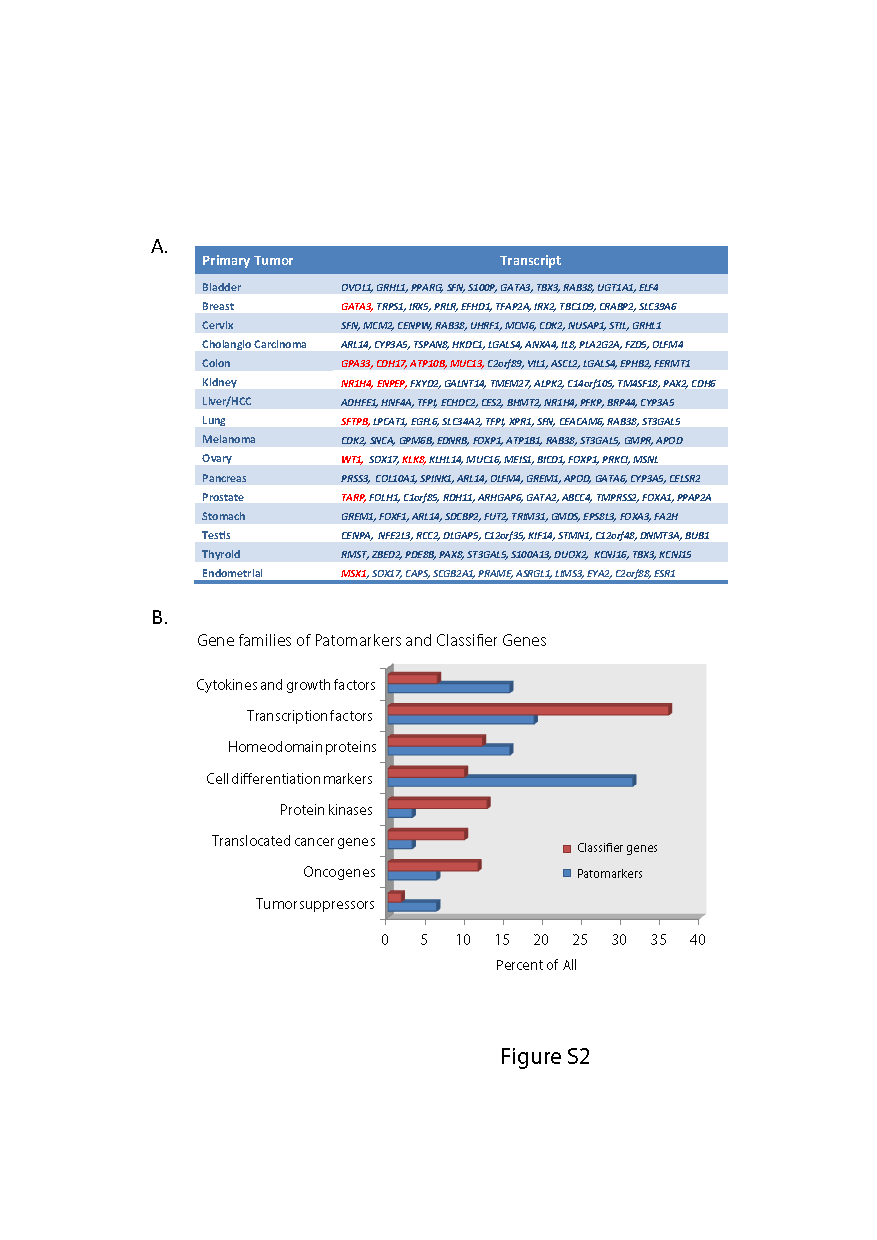

Supplement: Additional file 4: Figure S2. — Gene families and tumormarkers A. The 10 most selective transcripts in the 641 classifier for each tumor class. Transcripts were selected by comparison of the indicated tumor class with all other primary cancers and normal tissues and the ten transcripts exhibiting the lowest p value are shown. Due to the small sample number in cholangiocarcinoma and hepatocellular carcinoma, transcripts exhibited p < 0.01 and 10−4, respectively. For all other groups p < 10−10. Genes marked in red were considered to be almost exclusive to the class. B. Comparison of gene families in the patomarker and 641 classifier gene sets. Gene symbols of the two gene sets were submitted to the Brookhaven gene set enrichment analysis molecular signatures database (http://www.broadinstitute.org/gsea/msigdb) and examined with the Gene Families feature. The diagram shows the percentage of transcripts in each functional category. The 641 classifier transcripts are shown in red bars and the patomarkers are shown in blue bars. [file 12885_2015_1128_MOESM4_ESM.tiff]

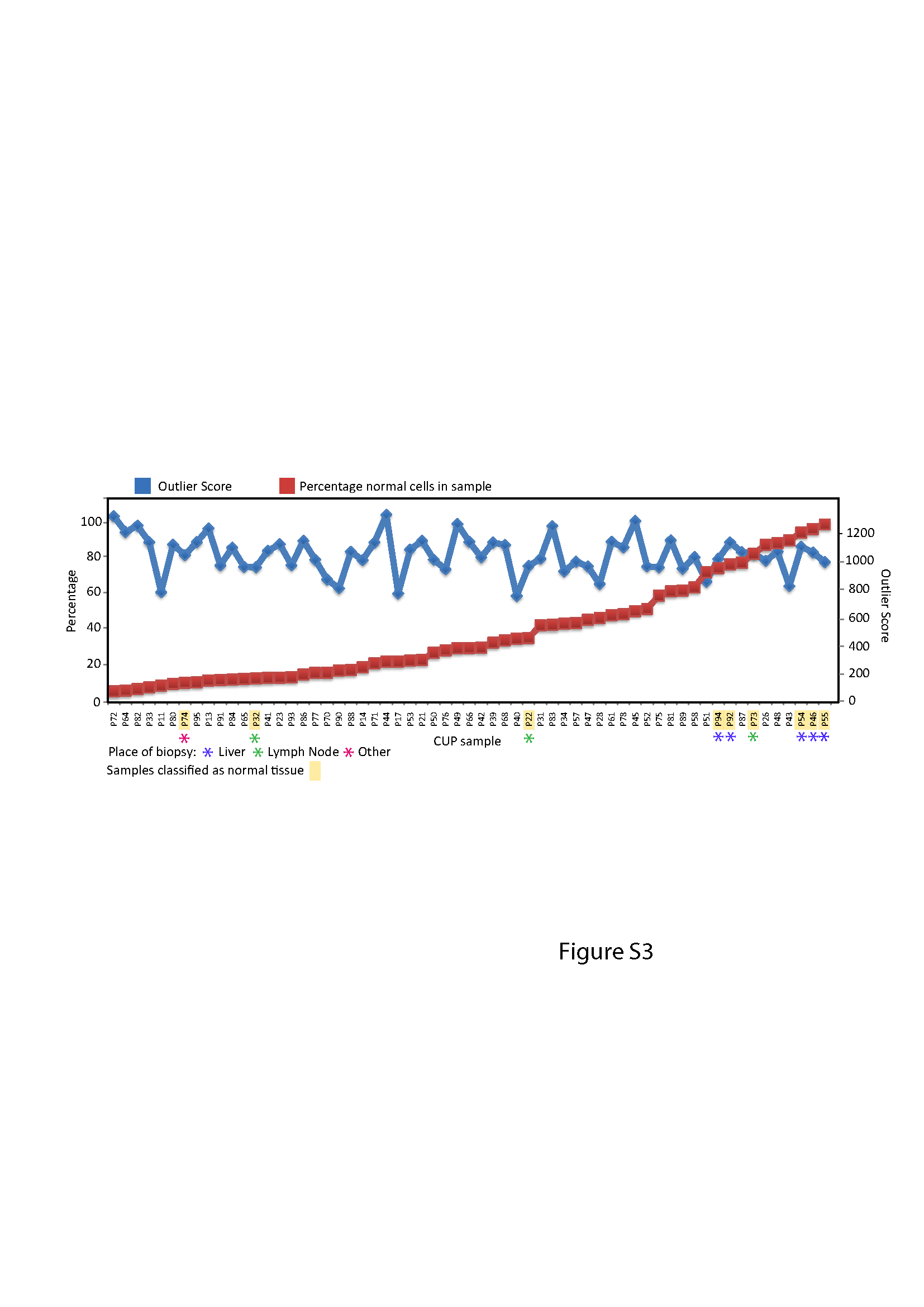

Supplement: Additional file 5: Figure S3. — Percentage of normal cells in CUP biopsies and QDA derived outlier scores. The percentage of normal cells in the CUP biopsies was determined by comparing the levels of liver (APOA2, ALB), muscle (ACTA1), lymph node (IGJ, IGHA1, IGKV3-20) and skin (KRT2, TYRP1) specific transcripts in the samples with the expression of the transcripts in corresponding normal tissues where the biopsies were obtained. The number indicates the CUP sample code and samples labeled in yellow were classified as normal tissue. The blue graph shows the corresponding outlier score generated by the QDA analysis. [file 12885_2015_1128_MOESM5_ESM.tiff]

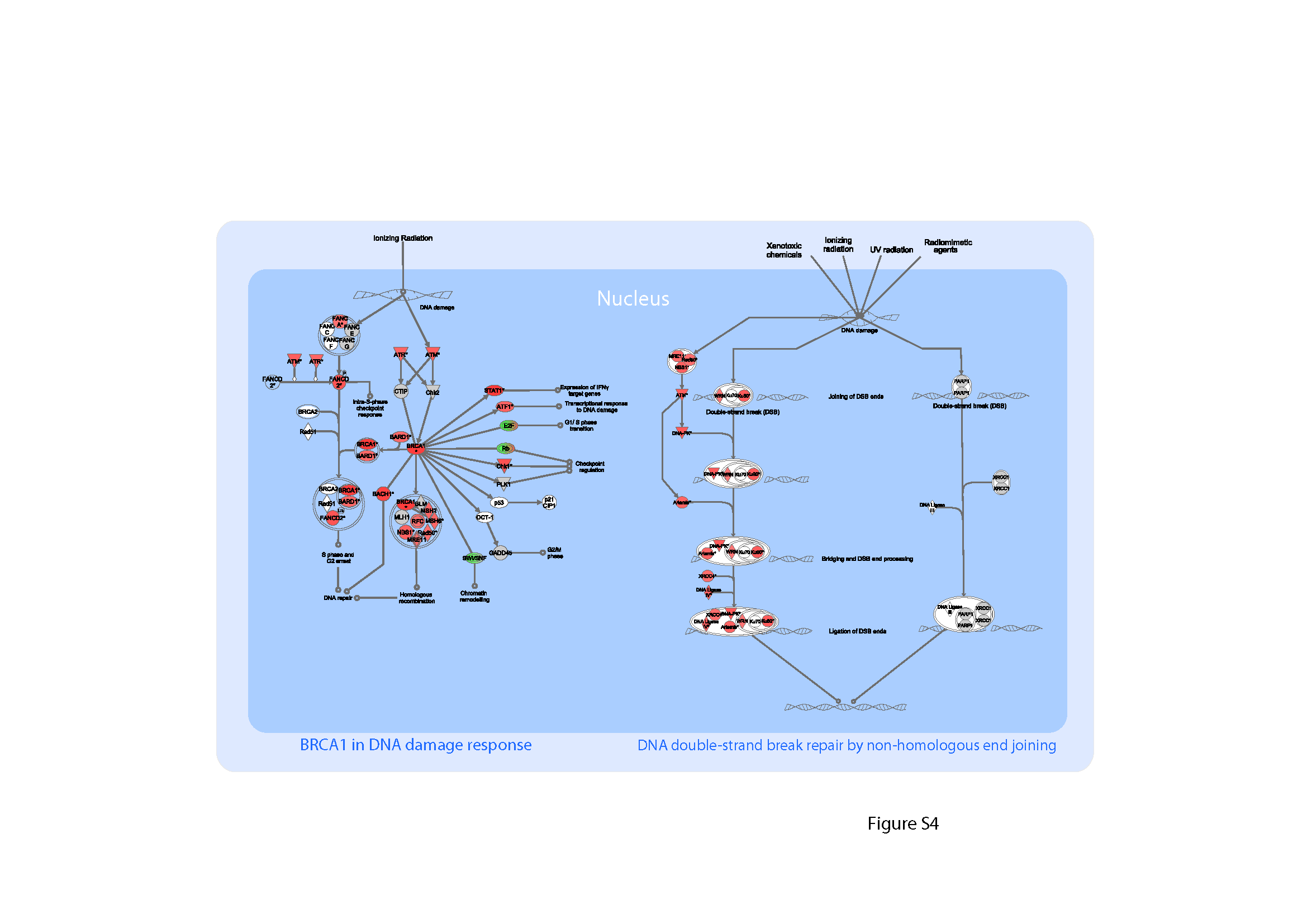

Supplement: Additional file 7: Figure S4. — Schematic representation of CUP enriched BRCA1 DNA damage response and non-homologous end joining repair networks. Diagrams were generated by the Ingenuity software (Ingenuity systems, USA). Up-regulated factors are indicated in red. [file 12885_2015_1128_MOESM7_ESM.tiff]
