# Supplementary material for: Cancers of unknown primary origin (CUP) are characterized by chromosomal instability (CIN) compared to metastasis of know origin
Source: BMC Cancer. 2015 Mar 19;15:151. doi: 10.1186/s12885-015-1128-x (PMC4404593; doi:10.1186/s12885-015-1128-x)
Supplement: Additional file 3: Table S2. — Prediction of tumor classes with the 428 and 641 classifiers. [file 12885_2015_1128_MOESM3_ESM.pdf]

Tumor Class	428 classifier Training set		428 classifier Test set		641 classifier Combined set	
	Samples (n)	Correct (%)	Samples (n)	Correct (%)	Samples (n)	Correct (%)
Bladder	40	77.5	5	100.0	45	84.4
Breast	260	95.8	52	84.6	312	94.2
Cervix	21	71.4	15	100.0	36	80.6
Cholangiocarcinoma	5	40.0	4	0	9	22.2
Colon/rectum	275	96.0	96	86.5	371	94.6
Hepatocellular carcinoma	6	66.7	5	100.0	11	90.9
Kidney	191	97.4	62	88.7	253	96.4
Lung	95	90.5	64	96.9	159	92.5
Malignant melanoma	13	76.9	46	89.1	59	91.5
Ovary	121	90.1	183	91.8	304	90.5
Pancreas	19	78.9	39	84.6	58	89.7
Prostate	60	98.3	18	77.8	78	93.6
Stomach/cardia	18	72.2	21	66.7	39	84.6
Testis	6	100.0	5	80.0	11	90.9
Thyroid	47	87.2	14	78.6	61	82.0
Uterus	122	90.2	12	50.0	134	85.8
Normal	167	90.4			268	88.8
Overall	1466	92.2	641	87.4	2208	91.3
Primary tumours	1299	92.4	391	90.3	1690	92.3
Metastases	-	-	250	82.8	250	86.8
Normal tissue	167	90.4	101	72.2	268	88.8

Table S2. Prediction of tumor classes with the 428 and 641 classifiers.
