# Supplementary material for: Cancers of unknown primary origin (CUP) are characterized by chromosomal instability (CIN) compared to metastasis of know origin
Source: BMC Cancer. 2015 Mar 19;15:151. doi: 10.1186/s12885-015-1128-x (PMC4404593; doi:10.1186/s12885-015-1128-x)
Supplement: Additional file 6: Table S4. — Forty most up---(red) or down---regulated (blue) mRNAs in CUP compared to metastasis of known origin. Differentially expressed transcripts, were retrieved by a class comparison between CUPs and metastasis The analysis was performed as a paired analysis with respect to the LDA predictions to eliminate differences related to the individual tumor classes. Metastases from endometrial, testis, prostate, melanoma and thyroid cancers were excluded from the analysis because no CUP had been allocated to these groups by the LDA. CUPs predicted as normal tissue were also excluded. [file 12885_2015_1128_MOESM6_ESM.pdf]

Probeset	Transcript	p-value	q-value	Probeset	Transcript	p-value	q-value
39854_r_at	*PNPLA2*	2.08E-21	5,17E-18	1557081_at	*RBM25*	2.67E-19	3,18E-16
226667_x_at	*EPN1*	3.34E-21	7,57E-18	230267_at	---	1.90E-19	2,31E-16
227404_s_at	*EGR1*	3.46E-21	7,57E-18	214149_s_at	*ATP6V0E1*	1.55E-19	1,93E-16
77508_r_at	*RABEP2*	3.68E-20	5,75E-17	242803_at	---	1.04E-19	1,35E-16
217240_at	*SIRPB1*	1.30E-19	1,65E-16	1556658_a_ε	---	9.82E-20	1,31E-16
234924_s_at	*ZNF687*	1.21E-18	1,16E-15	204970_s_at	*MAFG*	8.97E-20	1,23E-16
221956_at	*LRCH4*	2.87E-18	2,45E-15	237119_at	---	8.18E-20	1,15E-16
204104_at	*SNAPC2*	3.09E-18	2,52E-15	222728_s_at	*TAF1D*	7.96E-20	1,15E-16
229783_at	*AKAP13*	4.36E-18	3,31E-15	215450_at	*SNRPE*	5.66E-20	8,36E-17
241944_x_at	---	5.70E-18	4,16E-15	241845_at	---	5.57E-20	8,36E-17
239485_at	*CDH4*	5.78E-18	4,16E-15	1567213_at	*PNN*	3.53E-20	5,67E-17
214335_at	*RPL18*	6.36E-18	4,46E-15	215114_at	*SENP3*	2.84E-20	4,70E-17
233207_at	*DISC1*	1.28E-17	8,04E-15	200630_x_at	*SET*	1.28E-20	2,19E-17
208390_s_at	*GLP1R*	2.36E-17	1,37E-14	237716_at	---	7.57E-21	1,33E-17
1552504_a_ε	*BRSK1*	3.10E-17	1,73E-14	229399_at	*C10orf118*	7.34E-21	1,33E-17
1558048_x_ε	---	4.31E-17	2,29E-14	237383_at	---	5.00E-21	9,42E-18
206817_x_at	*CELF3*	4.44E-17	2,32E-14	244312_at	---	4.92E-21	9,42E-18
203617_x_at	*ELK1*	4.83E-17	2,46E-14	228471_at	*ANKRD44*	4.34E-21	8,78E-18
211659_at	*GPR135*	4.91E-17	2,46E-14	1559455_at	---	3.79E-21	7,96E-18
225254_at	*CCDC97*	5.65E-17	2,78E-14	242563_at	---	2.92E-21	6,94E-18
210780_at	*ESR2*	6.59E-17	3,19E-14	214052_x_at	*BAT2L2*	1.89E-21	4,93E-18
41387_r_at	*KDM6B*	7.00E-17	3,25E-14	237426_at	*SP100*	1.47E-21	4,03E-18
233322_at	*CD9*	8.32E-17	3,68E-14	239649_at	---	7.22E-22	2,19E-18
207282_s_at	*MYOG*	8.34E-17	3,68E-14	235701_at	---	5.83E-22	1,88E-18
238267_s_at	---	8.42E-17	3,68E-14	231199_at	---	3.13E-22	1,07E-18
237642_at	*C4orf42*	8.51E-17	3,68E-14	232529_at	*SP3*	2.34E-22	8,54E-19
203792_x_at	*PCGF2*	8.62E-17	3,68E-14	242772_x_at	---	2.26E-22	8,54E-19
215616_s_at	*KDM4B*	8.64E-17	3,68E-14	210231_x_at	*SET*	8.32E-23	3,50E-19
244541_x_at	---	1.04E-16	4,32E-14	243527_at	---	5.61E-23	2,56E-19
233756_at	---	1.17E-16	4,82E-14	208835_s_at	*LUC7L3*	5.19E-23	2,56E-19
208139_s_at	---	2.77E-16	1,05E-13	214313_s_at	*EIF5B*	4.91E-23	2,56E-19
1553130_at	*LOC652276*	2.96E-16	1,11E-13	201024_x_at	*EIF5B*	4.37E-23	2,56E-19
206217_at	*EDA*	3.06E-16	1,13E-13	233314_at	*PTEN*	3.85E-23	2,56E-19
231463_at	*CNTD1*	3.57E-16	1,26E-13	1568815_a_ε	*DDX50*	2.12E-23	1,65E-19
244656_at	*RASL10B*	3.83E-16	1,32E-13	214314_s_at	*EIF5B*	1.00E-23	9,13E-20
238371_s_at	---	4.31E-16	1,44E-13	237577_at	*PCNP*	1.85E-24	2,02E-20
1556206_at	---	4.75E-16	1,55E-13	238738_at	*PSMD7*	1.19E-24	1,62E-20
217509_x_at	*GRIK5*	5.31E-16	1,71E-13	242428_at	*DCUN1D1*	1.61E-25	2,94E-21
216292_at	---	5.91E-16	1,89E-13	201026_at	*EIF5B*	3.21E-26	8,77E-22
1559911_at	---	6.52E-16	2,06E-13	242427_at	*WAC*	3.12E-26	8,77E-22

Table S4. Forty most up- (red) or down-regulated (blue) mRNAs in CUP compared to metastasis of known origin. Differentially expressed transcripts, were retrieved by a class comparison between CUPs and metastasis The analysis was performed as a paired analysis with respect to the LDA predictions to eliminate differences related to the individual tumor classes. Metastases from endometrial, testis, prostate, melanoma and thyroid cancers were excluded from the analysis because no CUP had been allocated to these groups by the LDA. CUPs predicted as normal tissue were also excluded.
