# Supplementary material for: Cancers of unknown primary origin (CUP) are characterized by chromosomal instability (CIN) compared to metastasis of know origin
Source: BMC Cancer. 2015 Mar 19;15:151. doi: 10.1186/s12885-015-1128-x (PMC4404593; doi:10.1186/s12885-015-1128-x)
Supplement: Additional file 8: Table S3. — LDA predictions in CUP. [file 12885_2015_1128_MOESM8_ESM.pdf]

Tumor	Number	Percentage
Bladder	1	2
Breast	7	15
Cholangiocarcinoma	9	19
Cervix	3	6
Colon	5	10
HCC	1	2
Kidney	3	6
Lung	6	13
Ovary	4	8
Pancreas	4	8
Stomach	5	10

Supplemental Table 3. LDA predictions in CUP.
